# Polyethylene glycol-functionalized poly (Lactic Acid-*co*-Glycolic Acid) and graphene oxide nanoparticles induce pro-inflammatory and apoptotic responses in *Candida albicans*-infected vaginal epithelial cells

**DOI:** 10.1371/journal.pone.0175250

**Published:** 2017-04-03

**Authors:** R. Doug Wagner, Shemedia J. Johnson, Zhixia Yan Danielsen, Jin-Hee Lim, Thilak Mudalige, Sean Linder

**Affiliations:** 1 Microbiology Division, National Center for Toxicological Research, Jefferson, Arkansas, United States of America; 2 Division of Clinical Pharmacology IV, Office of Clinical Pharmacology, Office of Translational Sciences, Center for Drug Evaluation and Research, Food and Drug Administration, White Oak, Maryland, United States of America; 3 NCTR/ORA Nanotechnology Core Facility Jefferson, Arkansas, United States of America; National Institutes of Health, UNITED STATES

## Abstract

Mucous-penetrating nanoparticles consisting of poly lactic acid-*co*-glycolic acid (PLGA)-polyethylene glycol (PEG) could improve targeting of microbicidal drugs for sexually transmitted diseases by intravaginal inoculation. Nanoparticles can induce inflammatory responses, which may exacerbate the inflammation that occurs in the vaginal tracts of women with yeast infections. This study evaluated the effects of these drug-delivery nanoparticles on VK2(E6/E7) vaginal epithelial cell proinflammatory responses to *Candida albicans* yeast infections. Vaginal epithelial cell monolayers were infected with *C*. *albicans* and exposed to 100 μg/ml 49.5 nm PLGA-PEG nanospheres or 20 μg/ml 1.1 x 500 nm PEG-functionalized graphene oxide (GO-PEG) sheets. The cells were assessed for changes in mRNA and protein expression of inflammation-related genes by RT-qPCR and physiological markers of cell stress using high content analysis and flow cytometry. *C*. *albicans* exposure suppressed apoptotic gene expression, but induced oxidative stress in the cells. The nanomaterials induced cytotoxicity and programmed cell death responses alone and with *C*. *albicans*. PLGA-PEG nanoparticles induced mRNA expression of apoptosis-related genes and induced poly (ADP-ribose) polymerase (PARP) cleavage, increased BAX/BCL2 ratios, and chromatin condensation indicative of apoptosis. They also induced autophagy, endoplasmic reticulum stress, and DNA damage. They caused the cells to excrete inflammatory recruitment molecules chemokine (C-X-C motif) ligand 1 (CXCL1), interleukin-1α (IL1A), interleukin-1β (IL1B), calprotectin (S100A8), and tumor necrosis factor α (TNF). GO-PEG nanoparticles induced expression of necrosis-related genes and cytotoxicity. They reduced autophagy and endoplasmic reticulum stress, and apoptotic gene expression responses. The results show that stealth nanoparticle drug-delivery vehicles may cause intracellular damage to vaginal epithelial cells by several mechanisms and that their use for intravaginal drug delivery may exacerbate inflammation in active yeast infections by increased inflammatory recruitment.

## Introduction

Vaginal yeast infection is a prevalent disease that affects many women during their reproductive years. The infection induces localized inflammation of the affected skin areas and the tissues of the vaginal canal. The inflammation induced by *C*. *albicans* infection is responsible for the morbidity of the disease. Vaginal yeast infections are treated with antifungal drugs and probiotic bacterial preparations, which reduce the inflammation.

The predominant cell type in the wall of the vaginal canal is the vaginal epithelial cell. We have shown that cultured vaginal epithelial cells (VEC) respond to yeast infections by activation of toll-like receptors which initiate intracellular signal transduction pathways that can induce production and secretion of pro-inflammatory cytokines [1]. Recent studies have shown that heat-labile molecules from vaginal epithelial cells recruit inflammatory cells to the site of infection [2]. In the vaginal tissue, those cytokines recruit immune cells from other parts of the body that cause the symptoms of inflammation at the site of infection.

Vaginal epithelial tissues are exposed to infectious agents and FDA-regulated products used for contraception and prevention of sexually transmitted diseases. Drugs, such as microbicides and protease inhibitors for HIV prophylaxis, that can be applied with conventional intravaginal drug delivery systems using gels or vaginal rings for prevention and treatment of sexually transmitted diseases, have limited effectiveness because of poor distribution and retention in the vaginal tissues [3, 4]. This factor, as well as the need for safe and simple patient self-administration, has prompted research and development of nanoparticle-based drug delivery systems that penetrate vaginal tissues.

Nanoparticles are particulate materials in size ranges from 1 to 100 nm that have physical properties that make them intriguing additives for enhanced delivery of drugs; however, some forms can be inflammatory because of the same properties that make them desirable as drug carriers, e.g., their unique oxidative properties and propensity for cellular internalization [5]. Polymeric nanoparticles can provide controlled release of microbicide, but must have surfaces modified to decrease adherence to mucous that washes them away from the vaginal mucosa [6]. Polymeric nanoparticles were able to deliver small-interfering RNA molecules into epithelial cells of mice after intravaginal delivery, and they did not induce inflammatory responses, compared to lipid-based delivery vehicles [7]. An example of another intravaginal drug-delivery nanotechnology product has been tested in a recent human drug trial; a microbicidal chemical that has properties that allow it to form dendrimer nanoparticles was prepared and given to women by the vaginal route of administration [8]. It was evaluated for the ability to inactivate HIV and to cause irritation to the vaginal tracts of the subjects. The product was tolerated by the subjects and could inhibit viral infection of cell cultures after it was recovered from the subjects. Some of the women in the trial reported adverse events, including vaginal *C*. *albicans* infections, which was not significantly different than placebo controls [9].

Other forms of nanoparticle drug delivery are also of interest. The PLGA polymers are used in medical devices because they are biodegradable and non-toxic. Thus, nanoparticles made of PLGA have attracted attention as potential carriers of drugs in the body. A major application for these drug carriers is to provide a persistent controlled-release dose of microbicidal drug in the vaginal tract to inhibit sexually transmitted infections [6, 10]. Inflammatory responses to PLGA nanoparticles in rodent models have been described, which implies that they might affect pro-inflammatory responses by vaginal tissues to *C*. *albicans* infections [10].

Another possible material for drug-delivery nanoparticles is graphene oxide, which is cytotoxic but it can be functionalized to limit toxicity [11]. A similar material, multi-walled carbon nanotubes, activates NLR family pyrin domain containing 3 (NLRP3) inflammasomes in lung fibroblast cells, with increased production of IL1B, IL18, IL8, depolarized mitochondrial membrane potential, increased reactive oxygen intermediate production, and lipid peroxidation [12]. Although PLGA, graphene, and polyethylene glycol (PEG) are biocompatible in medical devices, as nanoparticles these vehicles have potential to initiate inflammation and may exacerbate the morbidity of active vaginal yeast infections. In order to provide insight into the mechanisms by which drug-delivery nanoparticles may affect *C*. *albicans*-induced production of inflammatory cytokines by VEC, we conducted an experimental study to measure the effects of PLGA-PEG nanospheres and GO-PEG sheets on mRNA and protein expression of inflammatory cytokines and signal transduction proteins by VEC stimulated with *C*. *albicans*. Using real-time quantitative PCR measurements of mRNA concentrations of cytokines and intracellular signaling molecules to measure gene expression changes in VEC caused by nanoparticles, it was possible to detect activation of cell stress and programmed cell death mechanisms. We also measured oxidative effects and DNA damage in VEC by nanoparticles that were additive to *C*. *albicans* effects.

## Materials and methods

### Materials

PLGA-PEG diblock copolymer nanoparticles were constructed by Phosphorex, Inc. (Hopkinton, MA, USA) with diameters of 116.2 ± 43.5 nm by solvent evaporation, and passed tests for sterility and low endotoxin content. Graphene oxide nanoparticles (1.1 X 500 nm, 60% carbon and 40% oxygen composition) with 5% PEG_5000_ covalently attached were constructed by NANOCS, Inc. (Boston, MA, USA). The nanoparticles were dispersed in genital tract secretions (GTS) medium [13] with ultrasonic agitation at 10°C for 4 minutes at a frequency of 37 kHz in an Elmasonic P30SE bath sonicator (Tovatech, LLC, Springfield, NJ, USA). Physical properties of nanoparticle solutions were assessed, including zeta potential, size, shape, and aggregation [14] using transmission electron microscopy, atomic force microscopy, and dynamic light scattering zeta potential determinations. Particle size and zeta potential measurements were performed using a Malvern Instruments (Worcestershire, U.K.) Zetasizer Nano ZS DLS system. For particle size determination, approximately 70 μL of each sample was added to low-volume disposable cuvettes and allowed to equilibrate at 25°C for 120 s prior to analysis. Zeta potential measurements were performed using a disposable folded capillary cell from Malvern. Solutions of nanoparticles were tested for endotoxin content (E-Toxate, Sigma Chemical Company, St. Louis, MO, USA) to avoid false inflammatory responses. This procedure was used because nanoparticle optical interference can affect some photometric endotoxin assays [14]. Copolymer compositions of PLGA-PEG nanoparticles [15] were measured at 10 μg/ml concentrations in dimethyl sulfoxide-d_6_ (Sigma) with a 500 MHz NMR spectrometer.

### Microbial strains and human cell line growth conditions

*Candida albicans* B311 (ATCC 32354) was grown aerobically in Sabouraud’s dextrose broth (Thermo Fisher, Houston, TX, USA) at 37°C, as previously described [1]. The vaginal epithelial cell (VEC) line VK2 (E6/E7) (ATCC CRL-2616) is a model of the vaginal epithelium that responds to *C*. *albicans* infection by secretion of inflammatory cytokines [1]. Genetic identity of the cells was validated by the ATCC Cell Line Authentication Service through Promega Corporation (Madison, WI, USA). Culture samples and media were tested for *Mycoplasma* spp. contamination with a polymerase chain reaction (PCR) assay (Mycoplasma Detection Kit, Southern Biotech, Inc., Birmingham, AL, USA). Use of the VEC cell line was approved by the Research Involving Human Subjects Committee of the U.S. Food and Drug Administration. The VEC were seeded at a concentration of 1 X 10^5^/ml and grown to 70% confluence on 35 mm^2^ polyester culture well inserts [16] in serum-free keratinocyte medium (K-SFM) containing 5 ng/ml recombinant epidermal growth factor and 50 g/ml bovine pituitary extract (Invitrogen Corporation, Grand Island, NY, USA) and 1 X 10^−9^ M 17β-estradiol [17]. In previous studies with the cell line, we did not observe physical damage to the VEC by *C*. *albicans* infection [1]; however, damage to VEC by nanoparticles in the present study was detected by release of lactate dehydrogenase from the cells. The cells were grown at 37°C with a 10% CO_2_ atmosphere and 100% humidity. GTS medium [13] was used on the apical side of the VEC layers for cell contact with nanoparticles and *C*. *albicans* to simulate the environment of the vaginal lumen.

### Experimental design

The *C*. *albicans* challenge of VEC cultures is a model used in previous studies [1, 18]. The apical GTS medium side of the cell monolayers was challenged with 2 X 10^6^ CFU/well of an overnight Sabouraud dextrose broth (Thermo Fisher) culture of the virulent *C*. *albicans* strain. After 18 hours incubation, the cultures were treated with 2.5 μg/ml amphotericin-B (Sigma) and the microorganisms were washed off the epithelial cells. The VEC were recovered with a non-enzymatic cell dissociation buffer (Corning Cell Stripper^®^, Thermo Fisher) and RNA was extracted from them or the cells were collected for other assays. In these experiments, the VEC were grown to 70% confluence and exposed to 20 μg/ml GO-PEG nanoparticles [11, 19] or 100 μg/ml PLGA-PEG nanoparticles [20] for 2 hours followed by challenge with *C*. *albicans*. PLGA-PEG nanoparticle dosages were based on experiments showing linear uptake kinetics at 100 μg/ml concentrations of PLGA nanoparticles by vascular smooth muscle cells [16].

### Cyotoxicity assay

Cytotoxicity, determined as membrane damage to VEC, was assessed by release of LDH into the basolateral K-SFM growth medium using a commercial assay kit (CytoTox-ONE, Promega). This assay quantified the change in damage to the VEC monolayers by the fungus or as a result of treatment with the nanoparticles. This assay and the other assays using fluorescence intensity or chemiluminescence detection systems were tested with PLGA-PEG and GO-PEG nanoparticles added to the control groups and tested with reagents alone, to assure that they were not interfering with detection [21].

### Real-Time quantitative reverse-transcription polymerase-chain-reaction (RT-qPCR) gene expression profiling

Custom RT^2^ Profiler^TM^ RT-qPCR array applications from Qiagen-SABiosciences (Fredrick, MD, USA) were used to assess expression of mRNA for 178 genes listed in S1 Table, which may be involved in the response of VEC to contact with *C*. *albicans*. Total cellular RNA from VEC monolayers was isolated using RNeasy Protect^®^ total RNA isolation kits (Qiagen, Inc., Valencia, CA, USA), as previously described [1]. Results of the RT-qPCR array experiments were analyzed according to the manufacturer’s instructions to determine the key signal transduction pathways and immune system interaction genes involved in the probiotic effects on VEC stimulation by *C*. *albicans*.

### Flow cytometry assays

Flow cytometry was used to measure autophagy activation, quantified by Cyto-ID^®^ (Enzo Life Sciences, Farmingdale, NY, USA) dye binding to autophagosomes. Commercial assays were used for determinations of PARP cleavage and DNA damage (Apoptosis, DNA Damage and Cell Proliferation Kit^®^, BD Biosciences, San Jose, CA, USA), chromatin condensation (Nuclear-ID Green^®^, Enzo), mitochondrial membrane potential depolarization (MitoProbe DilC1 (5)^®^, Invitrogen), total reactive oxygen species production (CellROX Green^®^, Invitrogen, Carlsbad, CA, USA), protein aggregesomes (ProteoStat Aggregesome Detection kit^®^, Enzo), superoxide production (Total ROS/Superoxide Detection kit^®^, Enzo). These assays were run according to the manufacturer’s instructions in an Accuri C6 flow cytometer (BD Biosciences). The ratio of BAX/BCL2 production, indicative of early apoptosis stages, was determined using labelled antibodies from Santa Cruz Biotechnology (Santa Cruz, CA, USA). For the latter assay, VEC were rinsed with DPBS and then stained with fluorescent antibodies prior to analysis in an Accuri C6 flow cytometer (BD Biosciences). The flow cytometer was calibrated with SPHERO™ Rainbow Calibration Particles (Spherotech, Inc., Lake Forest, IL, USA). Density plots of measurements from side scatter versus the appropriate fluorescence channel were analyzed with polyhedral gates to determine the percentage of stained cells or median fluorescence values measured within assigned gates or quadrants.

### Fluorescence microscopy / high content analyses

A fluorescence imaging plate reader (Cytation 3, BioTek, Winooski, VT, USA) equipped with filter cubes for blue (435–485 nm), green (515–55 nm), and red (555–715 nm) fluorescence emission was used to image and measure the presence of fluorescent organelle-specific dyes in the cells. The plate reader function was used for measurements of dye-binding assays for cytotoxicity (CytoTox-ONE^®^) and oxidative stress (ROS-Glo^®^ and the GSH/GSSG-Glo Assay^®^, Promega). All assays were run according to the manufacturer’s instructions.

### Cytokine ELISA assays

Culture supernatants were analyzed with quantitative enzyme-linked immunoassays for IL1A, IL-1B, IL18, CXCL1, TNF, and S100A8 according to the manufacturer’s instructions (RayBiotech, Inc., Norcross, GA, USA).

### DNA damage and cellular oxidative damage assessment

Nanoparticles can cause DNA damage to human cells [22]. DNA damage in cells was assessed using an assay for γH2AX histone phosphorylation (Apoptosis, DNA Damage and Cell Proliferation Kit^®^, BD Biosciences). Nanoparticles can cause oxidative damage to cells that induces apoptosis [23]. Oxidative responses in VEC were measured with a Molecular Probes CellROX Green^®^ assay (Invitrogen) that detects an oxidation-sensitive dye bound to DNA in the cells by flow cytometry and also the ROS-Glo™ assay (Promega) for H_2_O_2_ measurements by high content analysis. Superoxide production was measured (Total ROS/Superoxide Detection kit^®^, Enzo) and the amount of oxidized glutathione was also measured (GSH/GSSG-Glo Assay™, Promega), as a measure of oxidative damage responses in the cells.

### Assessment of mitochondrial damage

Nanoparticle drug-delivery vehicles may disturb mitochondrial health, which can induce apoptosis by the intrinsic caspase pathway or other cell death mechanisms. To evaluate the effects of nanoparticles on mitochondria, changes in mitochondrial membrane potential (ΔΨm) were measured, which often depolarizes prior to apoptosis. Molecular Probes™ DilC1(5) dye, that loses fluorescence intensity at 658 nm when the mitochondria depolarize, was used to measure depolarization of ΔΨm [24]. Carbonyl cyanide 3-chlorophenylhydrazone (CCCP) was used as a positive control treatment for ΔΨm depolarization.

### Evaluation of cell death responses

Induction of cellular apoptosis was assayed as an increase in the ratio of BCL2-associated X protein (BAX) to B-cell CLL \ lymphoma 2 (BCL2) protein expression, which was measured by flow cytometry with reagents from Santa Cruz Biosciences. Other forms of cell death (pyroptosis and necrosis) can be differentiated from apoptosis using inhibitors of cell membrane permeabilization (5 mM glycine) and the apoptosis-related cysteine peptidase 1 (CASP1) activation inhibitor 100 μM benzyloxycarbonyl-Tyr-Val-Ala-Asp(Omethyl)-fluoromethyl ketone [25]. A fluorometric assay was used to measure the relative amounts of activation of CASP1 with the RayBio^®^ Caspase-1/ICE Fluorometric Assay kit (RayBiotech).

### Assessment of protein damage stress

Autophagy is a process that cells use to digest internal components and engulfed particles to recover nutrients. It is also a process that occurs during some forms of cell death or as a way to prevent cell death [26]. Activation of autophagy was assessed with a fluorescent dye (Cyto-ID™ (Enzo) that is quenched unless the dye is incorporated into cellular membranes formed by fusion of autophagosomes and lysosomes. Chloroquine (Sigma) was used as a specific inhibitor of lysosomal acidification to assess the autophagy initiation pathways involved. Cells can be induced to enter apoptosis when they are under the stress of the unfolded protein response (UPR). We used a red fluorescent rotor dye ProteoStat™ (Enzo) to stain protein aggregesomes and measured their relative concentrations by flow cytometry. A positive control supplied in the kit, MG-132, was used as an inducer of aggregesome formation because it inhibits proteasome activity.

### Data analysis

The mean fold differences in expression of genes detected by the RT-qPCR arrays from 6 experiments were compared for statistical significance by Repeated Measures One-Way Analysis of Variance (ANOVA) and Newman-Keuls post tests using Prism v. 6.0 software (GraphPad Software, San Diego, CA, USA). Nonparametric Kruskal-Wallis tests with Dunn’s Multiple Comparisons tests were applied to analyze results from flow cytometry analysis of phenotypic expression of the genes affected by the experimental treatments. Statistical significance was defined by a P<0.05. The number of samples per group was determined to be 6 in order to have 80% power of detecting a mean 0.92-fold change in expression of mRNA [27].

## Results

### Cell culture validation

The genetic identity of the VK2(E6-E7) cell line was verified by the Cell Line Authentication Service (Promega). The cell cultures were verified to be free of *Mycoplasma* spp. contamination by PCR analysis (S1 Fig).

### Characterization of nanoparticles

**Fig 1:**
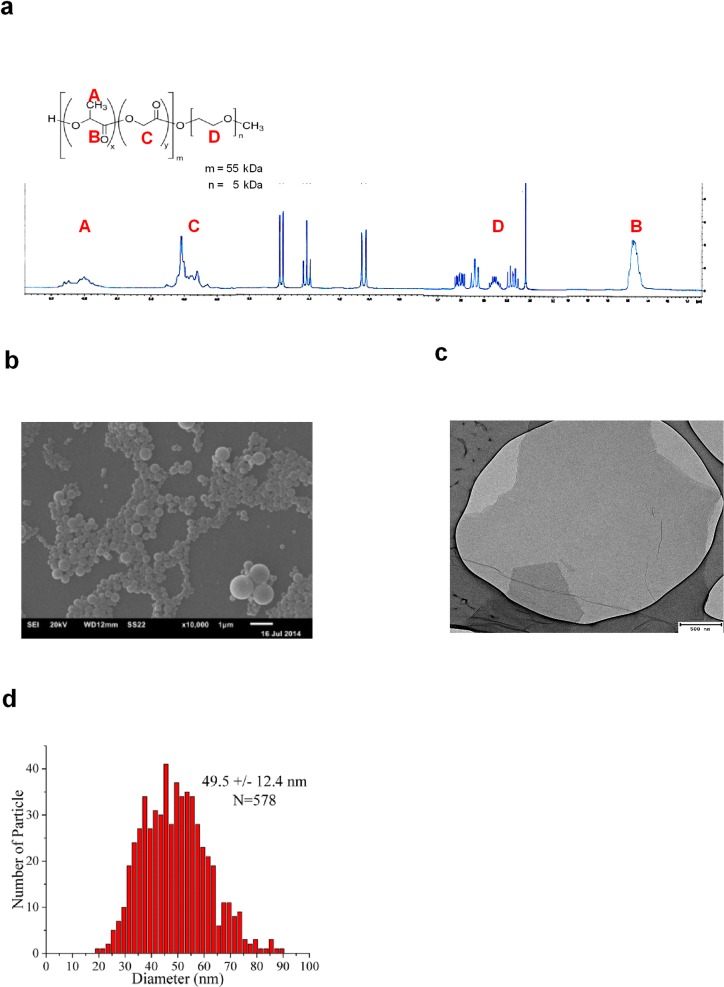
Synthesis and characterization of drug-delivery nanoparticles. (a) Structure of PLGA-PEG diblock copolymer for construction of 116 nm particles and ^1^H-NMR analysis of synthesized nanoparticles. The scan shows lactide methine quartets at 5.2 ppm (peak A), lactide methyl doublets at 1.5 ppm (peak B), glycolide proton singlets at 4.9 ppm (peak C), and polyethylene glycol methylene singlets at 3.5 ppm (peak D) characteristic of PLGA-PEG. (b) Scanning electron micrograph of PLGA-PEG nanoparticles. (c) Scanning electron micrograph of graphene oxide–polyethylene glycol nanoparticles. (d) Size distribution of PLGA-PEG nanoparticles determined by transmission electron microscopy.

Phosphorex, Inc. synthesized 116 nm PLGA-PEG nanoparticles, which were subsequently analyzed at the author’s facility for composition by nuclear magnetic resonance spectroscopy and characterized for size, shape, zeta potential, and polydispersity index with electron microscopy, atomic force microscopy, particle tracking analysis, and dynamic light scattering. The concentration of PEG in PLGA-PEG nanospheres was estimated to be 17 mol% by NMR from resonance integrations of lactide methine and methyl protons, glycolide methylene protons, and PEG methylene protons in the polymer structure (Fig 1A). Average size distributions of the PLGA-PEG nanoparticles were estimated to be 49.5 nm by transmission electron microscopy (TEM), 116 nm by nanoparticle tracking analysis, and 182 nm by dynamic light scattering analysis (Fig 1B). Scanning electron microscopy shows the size distributions and shapes of PLGA-PEG (Fig 1B) and GO-PEG nanoparticles used in this study (Fig 1C). The size distribution of PLGA-PEG nanoparticles measured by TEM (the most reliable technique for polymeric nanoparticles) is shown in Fig 1D. Dynamic light scattering analysis reported similar Mean ± SEM polydispersity indices of 0.433 ± 0.020 for PLGA-PEG nanoparticles in water, 0.581 ± 0.034 for PLGA-PEG in GTS medium, and 0.452 ± 0.065 for GO-PEG in GTS medium. The Mean ± SEM zeta potentials of the nanoparticles were of -20.93 ± 0.21 mV for PLGA-PEG nanoparticles in water, -1.47 ± 0.13 mV for PLGA-PEG in GTS medium, and -9.47 ± 0.22 mV for GO-PEG in GTS medium were significantly different from each other at P<0.05 by ANOVA.

### Cytotoxicity, DNA damage and apoptosis induction by *C*. *albicans* and nanoparticles

**Fig 2:**
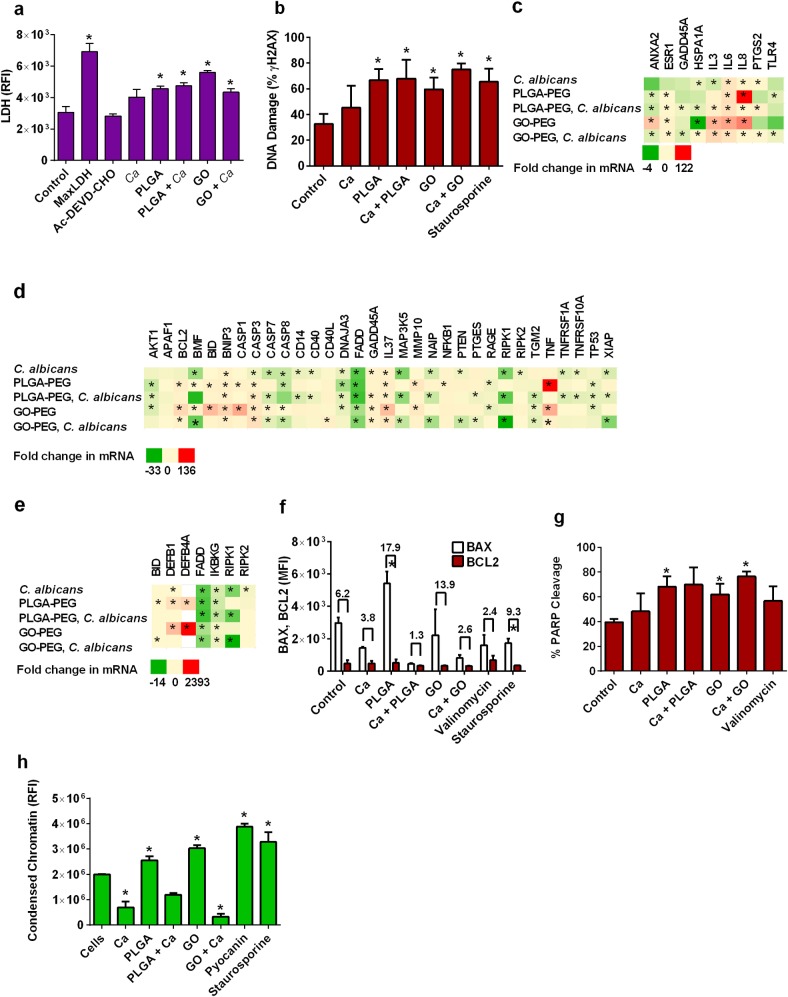
Cytotoxicity of nanoparticles to vaginal epithelial cells and phenotypic measurement of apoptosis mechanisms. (a) Cytotoxicity was measured as amounts of lactate dehydrogenase (LDH) released into the medium from VEC. (b) DNA stress was measured as % accumulation of phosphorylated nucleosome _Ɣ_H2AX in VEC. (c) Heatmaps illustrate how infection of VEC with *C*. *albicans* and exposure to nanoparticles induced changes in mRNA expression of genes associated with the stress of DNA damage and repair, (d) apoptosis, or (e) necrosis. (f) Increased ratio of BAX to BCL2 proteins indicative of apoptosis activation was measured in VEC. (g) The amount of PARP cleavage, indicative of apoptosis activation, was measured as the % of positively stained VEC. (h) Nuclear chromatin condensation was compared in VEC as relative nuclear dye binding (fluorescence intensity). PLGA-PEG and GO-PEG nanoparticle controls showed no interference with assay detection. PLGA-PEG and GO-PEG nanoparticle controls showed that the materials did not interfere with fluorescent detection signals in these assays. Data are Mean ± SEM, n = 3. *Significantly different than the control cells without treatments, P< 0.05. Ac-DEVD-CHO, N-Acetyl-Asp-Glu-Val-Asp-al was a negative control, Ca = *Candida albicans*, PLGA = PLGA-PEG nanoparticles, GO = GO-PEG nanoparticles, Max LDH = maximum release of LDH positive control, MFI = median fluorescence intensity, RFI = relative fluorescence intensity.

The relative amounts of cytotoxicity, leading to cell death induced by *C*. *albicans*, PLGA-PEG, or GO-PEG nanoparticles were compared by measurement of the amount of lactate dehydrogenase released from the VEC. Both types of nanoparticles were cytotoxic to the VEC, compared to the fungus or no challenge treatment (Fig 2A). Cell death can also ensue from DNA damage, which was observed as histone H2AX phosphorylation in vaginal epithelial cells treated with either type of nanoparticles (Fig 2B). *C*. *albicans* treatment alone increased the mean of DNA damage measurements, but it was not statistically significant. PLGA-PEG and GO-PEG nanoparticles induced expression of genes associated with DNA damage/repair stress (Fig 2C). Programmed cell death, or apoptosis, is the process used by the human body to recycle cells when they need to be eliminated safely. *C*. *albicans* infection induced mostly anti-apoptotic gene expression responses in VEC, whereas PLGA-PEG and GO-PEG nanoparticles induced pro-apoptotic responses (Fig 2D). *C*. *albicans* infection did not induce expression of necrosis-associated genes, but nanoparticles did, especially GO-PEG (Fig 2E). Apoptosis is regulated by a signaling cascade of enzymes that provide entry points for control. Two of the control enzymes are BCL2, which inhibits apoptosis and BAX, which induces apoptosis and overrides BCL2 inhibition. The ratio of intracellular expression of these two proteins (measured by flow cytometry) was used for evaluation of the degree of apoptosis signaling in the cells. PLGA-PEG, but not *C*. *albicans* or GO-PEG increased the ratio of BAX and BCL2 proteins (Fig 2F). Activation of apoptosis can be measured by detecting cleaved PARP, which accumulates during middle and late-stage apoptosis as a result of CASP3 activity. The amount of PARP cleavage was increased by PLGA-PEG and GO-PEG (Fig 2G). As further evidence, an assay to measure late-stage nuclear chromatin condensation showed that GO-PEG nanoparticles induced this feature of apoptosis in VEC, but *C*. *albicans* suppressed this process in the cells (Fig 2H).

### *C*. *albicans* and nanoparticle induction of oxidative stress

**Fig 3:**
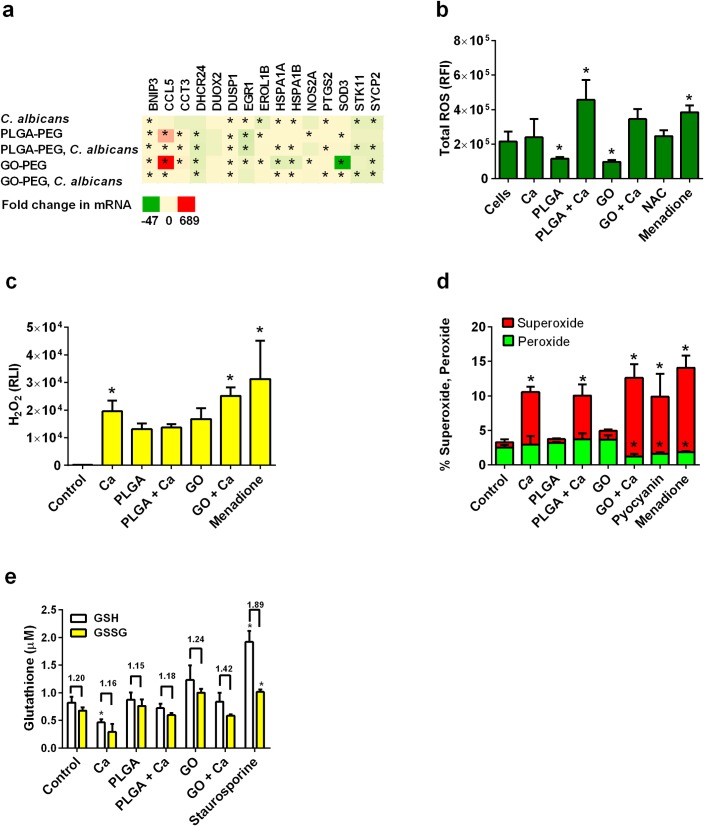
Phenotypic measurements of reactive oxygen intermediates produced by VEC. (a) This heatmap shows how the infection of VEC with *C*. *albicans* and exposure of these cells to nanoparticles induce changes in mRNA expression of genes associated with oxidative stress. (b) Total reactive oxygen intermediate production was detected in VEC measured as fluorescent dye binding. NAC treatment was an additional negative control. (c) H_2_O_2_ production was compared in VEC as activation of a chemiluminescent reagent. (d) Superoxide and peroxide production were measured in VEC as specific fluorescent dye binding. (e) Glutathione and oxidized glutathione concentrations were measured in VEC. The numbers above bars are the ratios of GSH/GSSG. PLGA-PEG and GO-PEG nanoparticle controls showed no interference with assay detection. PLGA-PEG and GO-PEG nanoparticle controls showed that the materials did not interfere with fluorescence or chemiluminescence detection signals in these assays. Data are Mean ± SEM, n = 3. *Significantly different than the control cells without treatments, P< 0.05. Ca = *Candida albicans*, GSH = reduced glutathione, GSSG = oxidized glutathione, NAC = N-acetyl-cysteine, RFI = relative fluorescence intensity, RLI = relative luminescence intensity.

Under various types of stress, cells will produce reactive oxygen intermediates. The treatments in this study induced oxidative stress response-associated genes in the cells with *C*. *albicans*, PLGA-PEG or GO-PEG, especially considering the increased expression of BCL2/adenovirus E1B 19 KDa interacting protein 3 (*BNIP3*), chemokine (C-C motif) ligand 5 (*CCL5*), and prostaglandin-endoperoxide synthase 2 (*PTGS2*) (Fig 3A). We observed indications that oxidative stress was imposed by the nanomaterials on VEC. In a comparison of overall reactive oxygen species (ROS) production, PLGA-PEG treatment with *C*. *albicans* induced ROS production, whereas the nanoparticles alone did not (Fig 3B). Individual types of reactive oxygen intermediates were produced at different levels in response to contact with PLGA-PEG, GO-PEG, and/or *C*. *albicans*. H_2_O_2_ production was induced by *C*. *albicans* and GO-PEG nanoparticles (Fig 3C). Superoxide production was significantly induced by the positive controls pyocyanin and menadione and by *C*. *albicans* with or without nanoparticles (Fig 3D). Increased production of oxidized glutathione (GSSG), or a decreased ratio of reduced glutathione (GSH) to GSSG, is an indicator of oxidative stress, which was observed in GO-PEG-treated VEC (Fig 3E).

### Mitochondrial stress was observed in PLGA-PEG and GO-PEG-treated cells

**Fig 4:**
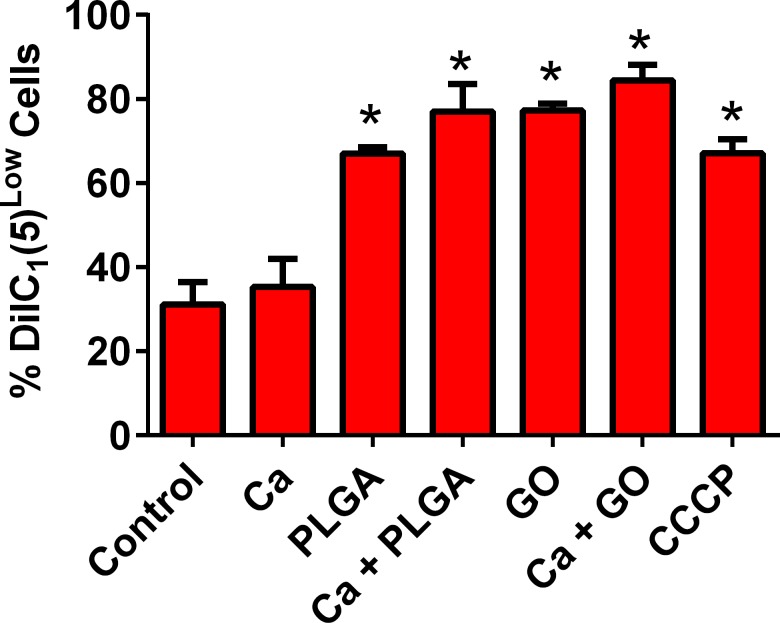
The nanoparticles induced membrane potential (ΔΨm) depolarization, an indication of mitochondrial damage. Mitochondrial ΔΨm depolarization was measured as Mean ± SEM % cells with reduced DilC1(5) dye accumulation in VEC. CCCP is a positive control. PLGA-PEG and GO-PEG nanoparticle controls showed that the materials did not interfere with fluorescence detection signals in this assay. *Significantly different than the control cells without treatments, P< 0.05, n = 3. Ca = *Candida albicans*, GO = GO-PEG nanoparticles, PLGA = PLGA-PEG nanoparticles.

Another cell stress mechanism is mitochondrial damage, in which membrane potential (ΔΨm) depolarization is an indication. An assay for mitochondrial stress uses fluorescence of the DilC1(5) dye that loses fluorescence intensity when the mitochondria depolarize, showed significant increases in depolarization of ΔΨm by PLGA-PEG and GO-PEG nanoparticles, with and without *C*. *albicans* (Fig 4).

### Autophagy and endoplasmic reticulum stress

**Fig 5:**
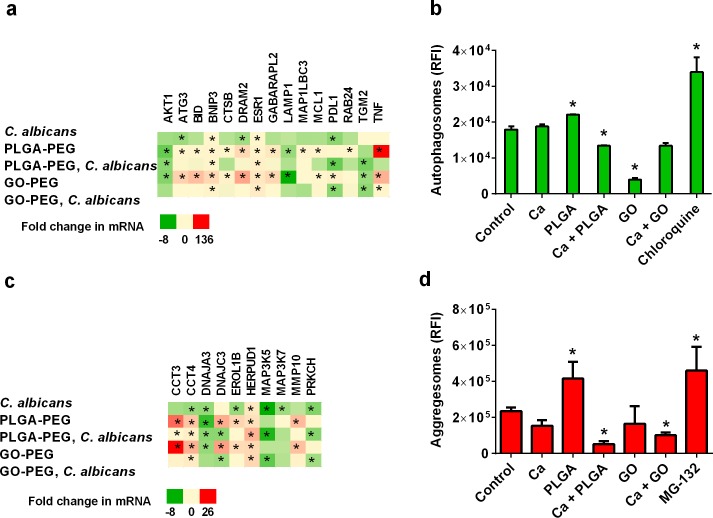
Endoplasmic reticulum stress was induced by nanoparticles. (a) This heatmap shows changes in expression of mRNA for genes associated with autophagy. (b) Production of autophagic vesicles was measured in VEC. Chloroquine was a positive control. (c) This heatmap shows changes in expression of mRNA for genes associated with endoplasmic reticulum or protein folding stress. (d) Accumulation of protein aggregesomes was measured in VEC. MG-132 was a positive control. PLGA-PEG and GO-PEG nanoparticle controls showed that the materials did not interfere with fluorescence detection signals in these assays. Data are Mean ± SEM, n = 3. *Significantly different than the control cells without treatments, P< 0.05. Ca = *C*. *albicans*; GO = GO-PEG nanoparticles; PLGA = PLGA-PEG nanoparticles; RFI = relative fluorescence intensity.

Autophagy is a process that cells use to digest internal components and engulfed particles to recover nutrients. It is also a process that occurs during some forms of cell death or as a way to prevent cell death. Cellular uptake of nanoparticles may stress endocytic trafficking in the cells and activate autophagy and endoplasmic reticulum (unfolded protein) stress. In this study, PLGA-PEG nanoparticles induced expression of autophagy-associated genes autophagy related 3 (*ATG3*), DNA damage regulated autophagy modulator 2 (*dram2*), GABA type A receptor associated protein like 2 (*GABARAPL2*), and RAB24, member RAS oncogene family (RAB24), (Fig 5A). GO-PEG induced expression of the autophagy modulators *ATG3*, *dram2* and *GABARAPL2*, (Fig 5A). Induction of the accumulation of autophagosomes in VEC was observed when treated with PLGA-PEG, but reduced accumulation of autophagosomes was observed in VEC with PLGA-PEG + *C*. *albicans* or GO-PEG nanoparticles (Fig 5B). Although autophagy-related genes were activated and autophagosome activity was slightly increased by PLGA-PEG nanoparticles, these results suggest autophagy activation may not be a contributor to the cell stress and programmed cell death induced by PLGA-PEG nanoparticles.

Endoplasmic reticulum stress may be the more likely source of cell stress induced by PLGA-PEG and GO-PEG nanoparticles. Both types of nanoparticles induced expression of genes involved in unfolded protein and endoplasmic reticulum stress responses (Fig 5C). Gene induction of chaperone containing TCP1 subunit 3 (*cct3*) and *cct4*, which code for proteins that prevent aggregesome formation [28], DNA J heat shock protein family (HSP 40) member C3 (*DNAJC3*) [29], and homocycteine inducible ER protein with ubiquitin like domain 1 (*HERPUD1*) [30] indicates activation of an unfolded protein response. Increased protein aggregesome formation in the cells shows that PLGA-PEG induced endoplasmic reticulum stress in vaginal epithelial cells (Fig 5D). Although statistical significance was not achieved by the GO-PEG nanoparticles, it is possible that they also induced aggregesome formation. The presence of *C*. *albicans* suppressed both the gene expression and physiological activities of aggregesome formation.

### Pyroptosis induction

**Fig 6:**
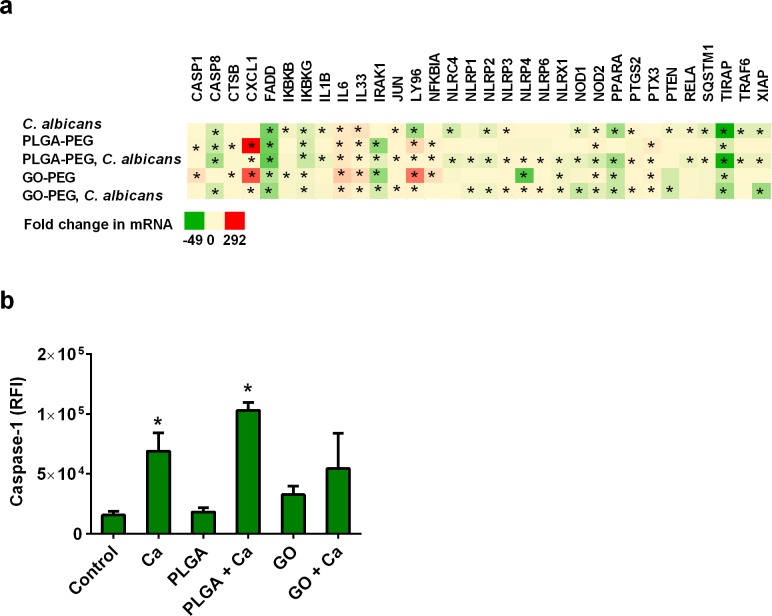
Pyroptosis and inflammasome activation. (a) Nanoparticles and *C*. *albicans* had effects on expression of mRNA associated with pyroptosis and inflammasome activation. (b) Relative comparison of caspase-1 activation was measured in VEC. PLGA-PEG and GO-PEG nanoparticle controls showed that the materials did not interfere with fluorescence detection signals in this assay. Data are Mean ± SEM, n = 3. *Significantly different than control cells without treatments, P<0.05. GO = GO-PEG nanoparticles; PLGA = PLGA-PEG nanoparticles; RFI = relative fluorescence intensity.

Increased expression of inflammasome genes can be an indication of pro-inflammatory responses in cells. Gene expression effects suggest that *C*. *albicans* infection favored pyroptosis via induction of NLRP3 inflammasomes with *casp1* and *nlrp3* expression slightly elevated (Fig 6A). The PLGA-PEG nanoparticles induced expression of *casp1*, nucleotide binding oligomerization domain containing 2 (*NOD*2), and pro-inflammatory genes (Fig 6A). The NOD2 protein is a receptor for muramyl dipeptides on microbial cell surfaces and can activate CASP1 and autophagy [31]. The GO-PEG nanoparticles induced expression of *CASP1* and pro-inflammatory genes (Fig 6A); however, changes in *CASP1* mRNA expression are not necessarily correlated with activation of CASP1 proteins. GO-PEG nanoparticle induction of necrosis via NOD2-associated inflammasomes was suggested by gene expression effects (Fig 6A). Pyroptosis is a form of programmed cell death involving activation of inflammasomes that activate CASP1, which proteolytically induces secretion of IL1B and IL18 in some cell types. *C*. *albicans* infection, but not nanoparticle exposure, was associated with cellular CASP1 activation, indicative of NLRP3-mediated pyroptosis (Fig 6B).

### Cytokine secretion

**Fig 7:**
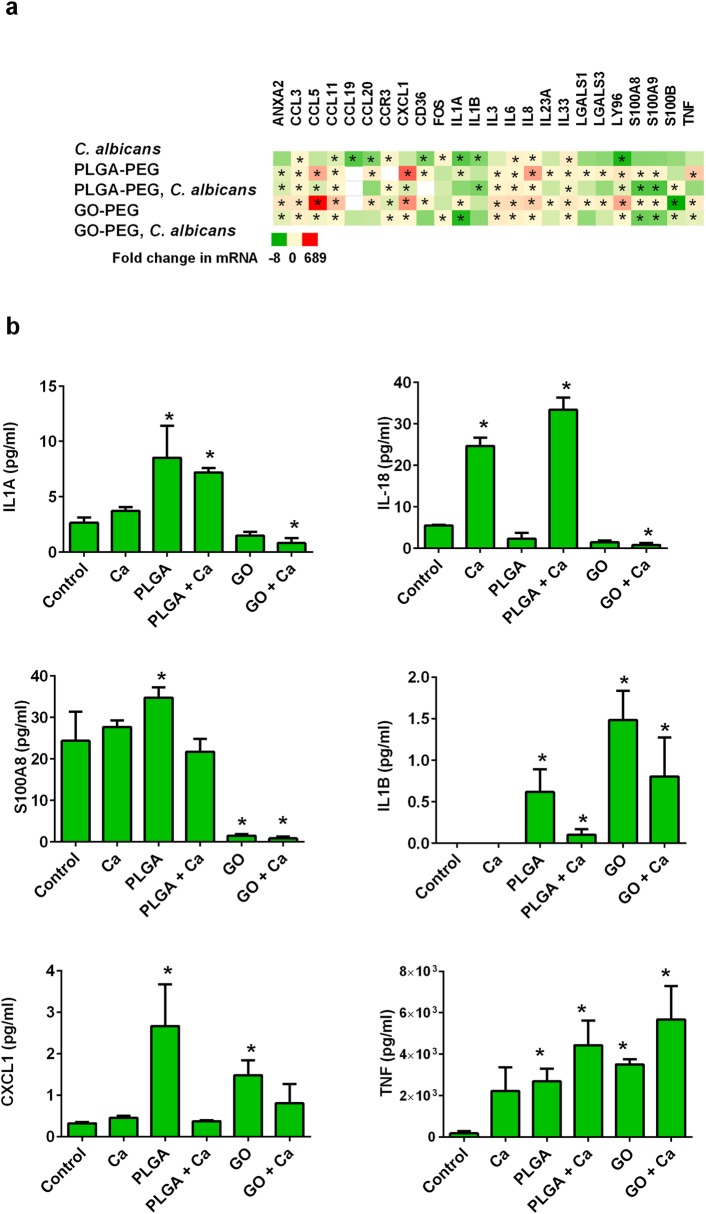
Nanoparticles and *C*. *albicans* modified inflammatory recruitment responses of VEC. (a) Changes in expression of genes associated with inflammatory recruitment were observed. (b) Cytokine concentrations secreted into the media by VEC were measured by enzyme linked immunosorbent assay. Data are Mean ± SEM, n = 3. *Significantly different than control cells without treatments, P<0.05. Ca = *Candida albicans*; GO = GO-PEG nanoparticles, PLGA = PLGA-PEG nanoparticles.

Vaginal epithelial cells mediate pro-inflammatory responses by secretion of specific mediators that attract inflammatory cells. Nanoparticles induced pro-inflammatory responses with increased mRNA expression of genes for cytokines, chemokines and alarmin molecules that recruit a broad range of leukocytes (Fig 7A). Secretion of some of these mediators into the culture media during challenge with nanoparticles and/or *C*. *albicans* was measured. Pyroptosis is characterized by cellular production of IL1B and IL18. Secretion of IL18, but not IL1B was induced by *C*. *albicans*, which is consistent with the pro-inflammatory nature of the infection (Fig 7B). Although IL1B secretion was not detected in control or *C*. *albicans*-treated cells, PLGA-PEG and GO-PEG nanoparticles induced production of IL1B (Fig 7B). Since HPV16 E6/E7-immortalized epithelial cells exhibit rapid proteolytic degradation of IL1B [32], the nanoparticles appeared to overcome this process, which also may account for the lack of *C*. *albicans*-induced IL1B production observed. Chemokine CXCL1 is an attractant chemokine for neutrophilic granulocytes, and its secretion by VEC was increased by challenge with PLGA-PEG and GO-PEG (Fig 7B). TNF production by VEC was induced by PLGA-PEG and GO-PEG nanoparticles, which is consistent with other results we observed indicating the pro-inflammatory and apoptosis-inducing effects of these nanomaterials (Fig 7B). PLGA-PEG also induced vaginal epithelial cells to secrete S100A8, which may have pro-inflammatory recruitment properties (Fig 7B). The production of S100A8 was significantly reduced in VEC treated with GO-PEG.

## Discussion

The availability of the intravaginal route of drug administration is an advantage to women that is widely exploited for self-administration of various treatments. It is especially fitting for delivery of drugs targeting the reproductive system, such as microbicides to prevent acquisition of sexually transmissible diseases. A complication of this strategy is that intravaginal drugs have to get beyond formidable barriers to contact the vaginal epithelium, but stealth, mucous-penetrating nanoparticles have been developed to overcome them [33]. This study was conducted to determine if such drug-delivery vehicles would pose a pro-inflammatory challenge to vaginal epithelial cells in the context of a *C*. *albicans* infection.

National drug regulatory agencies recommend intravaginal drug products be tested for irritation and inflammatory responses in the vaginal mucosa. They also recommend cytotoxicity information in cells likely to be affected by the product, such as vaginal epithelial cells, macrophages, and dendritic cells [34]. The research described in the present study revealed that cytotoxic mechanisms were induced in vaginal epithelial cells by PLGA-PEG and GO-PEG nanoparticles that could be used as drug carriers for vaginal microbicides. In accordance with a published guidance by the United States Food and Drug Administration [34], a PLGA-PEG nanoparticle formulation of an intravaginal biocide should be tested in a manner similar to what has been done in this work.

The nanoparticles were associated with increased cell death in our experiments. The PLGA-PEG and GO-PEG nanoparticles induced apoptotic mechanisms, but GO-PEG also induced other mechanisms of cell death, more associated with necrosis. The shape and dimensions of the GO-PEG nanoparticles could indicate an ability to cause physical damage to cell membranes that may account for the indications of necrosis. The induction of apoptosis by PLGA-PEG nanoparticles apparently occurred by the intrinsic apoptotic pathway, since mitochondrial depolarization was observed in the cells. Apoptosis responses to PLGA-PEG involved endoplasmic reticulum stress or unfolded protein response mediators and seemed to be modulated by autophagy. It is unclear what role autophagy plays in cell death mechanisms [26], so it may be a protective process in response to the nanoparticles. There may be a dose-dependent threshold in VEC that can tolerate a limited amount of nanoparticle uptake. The cytotoxicity of GO-PEG nanoparticles was greater than PLGA-PEG and was not associated with a weak activation of autophagy, which supports the idea that autophagy may have modulated PLGA-PEG-induced apoptosis, but was less protective in the necrosis-like cell death mechanisms activated by the GO-PEG. The GO-PEG nanoparticles appeared to interfere with autophagosome production, possibly by interactions with lipids and proteins and by oxidative reactions [35].

Interestingly, *C*. *albicans* infection suppressed apoptotic mechanisms, even in the presence of nanoparticles. *C*. *albicans* also induced oxidative stress not seen with nanoparticles. It induced the expected NLRP3 inflammasome-mediated activation of CASP1, and the cells secreted IL18, but not IL1B and did not induce significant release of LDH. Since the VEC were stimulated by the nanoparticles to secrete IL1B, the cells have that capability, but IL1B secretion was suppressed in the presence of *C*. *albicans*. We have observed a low IL1B response with these cells infected with *C*. *albicans* in a previous study [1]. The VK2 (E6/E7) cell line was originally made from normal human vaginal epithelial cells by transformation with E6 and E7 genes from human papilloma virus 16 [36]. Expression of E6 protein in the cells can induce proteasome activity on pro-IL1B molecules by interactions between E6 and host cell E6-AP ubiquitin ligase and TP53 [32]. However, E6 is also known to inhibit TP53 and induce expression of PI3K-AKT1-MTOR signal transduction pathways, which should also be considered in the interpretation of these results [37]. Thus, it may be difficult to extrapolate our results for IL1B effects to vaginal epithelial cells in vivo. We observed strong expression of *AKT1*, *MTOR*, and *TP53* mRNA by the cells in this study. Both *AKT1* and *TP53* expression were induced by *C*. *albicans*, PLGA-PEG, or GO-PEG treatments, suggesting that the effect of E6 expression was not an overriding factor on signal transduction that could affect autophagy and apoptosis signaling in this cell line. We still suggest that these results be considered in light of a possible E6 effect.

NLRP3 inflammasome activation is associated with mitochondrial degradation in bone marrow-derived macrophages [24]. In our present study, the activation of pyroptosis by *C*. *albicans* did not lead to mitochondrial ΔΨm depolarization of VEC. The PLGA-PEG and GO-PEG nanoparticles induced significant mitochondrial ΔΨm depolarization. PLGA-PEG also induced autophagy, which was suppressed by *C*. *albicans* infection, similar to the apoptosis effect described above. Future study should be applied to determine if the suppressive effect of *C*. *albicans* infection on cell stress responses in VEC are a feature of its role as a commensal organism or as an opportunistic pathogen.

PLGA-PEG appeared to induce NOD2 and IL1B secretion, but not CASP1 activation. A known pathogen-associated molecular pattern recognized by NOD2 is muramyl dipeptide exhibited on the surfaces of Gram-positive bacteria, such as *Streptococcus* pneumonia [38]. It is not clear why PLGA-PEG or GO-PEG induced *NOD2* expression in our present study, but it was associated with increased production of IL1A, IL1B, CXCL1, TNF, and S100A8. These results do show that the nanoparticles induced pro-inflammatory responses from the VEC. In macrophages, where inflammatory responses have been studied in more detail, NOD2 induces production of IL6, TNF, CXCL1, and chemokine (C-C motif) ligand 2 (CCL2) [39]. NOD2 regulation has been shown to be anti-inflammatory in colonic epithelial cells, where the nuclear factor of kappa light polypeptide gene enhancer In B-cells 1 (NFKB) signaling pathway that activates cytokine gene expression is suppressed at the level of the TNF receptor-associated factor 6, E3 ubiquitin protein ligase (TRAF6) and receptor (TNFRSF)-interacting serine-threonine kinase 1 (RIPK1) proteins [40]. Pro-inflammatory responses by VEC may be different than innate immune cells, and not anti-inflammatory as in colonic epithelial cells, but they may be important in recruitment of inflammation during yeast infections.

## Conclusions

*C*. *albicans* infection of VEC induced pro-inflammatory responses associated with oxidative stress and NLRP3-activated pyroptosis that led to IL18, but not IL1B secretion. The presence of nanoparticles representative of those proposed for intravaginal drug delivery exacerbated the oxidative responses. Both, PLGA-PEG and GO-PEG nanoparticles were cytotoxic in a medium representative of vaginal fluid. PLGA-PEG nanoparticles induced programmed cell death associated with NOD2 and intrinsic apoptosis, DNA damage, endoplasmic reticulum/protein-folding stress, and autophagy. GO-PEG nanoparticles induced intrinsic apoptosis and DNA damage. These results show that mucous-penetrating drug-delivery nanoparticles can cause cell stress and programmed cell death in VEC. Further study in vivo will be needed to assess whether pro-inflammatory responses of the vaginal epithelium to nanoparticle drug-delivery vehicles will be a problem in women with active vaginal yeast infections.

## Supporting information

S1 Fig *Mycoplasma* spp. assay. Evaluation of cell-line *Mycoplasma* spp. contamination status was measured by PCR. Lane 1 = Molecular Weight Marker, Lane 2 = negative control, Lane 3 = Positive control, Lane 4 = Sample of cells in culture media.
(TIF)

S1 Table List of genes analyzed by RT-qPCR. The National Center for Biotechnology Information (www.ncbi.nlm.nih.gov) gene symbols and reference sequence accession numbers are listed for the mRNA molecules that were quantified by RT-qPCR using DNA primers synthesized by Qiagen, Inc., based on the Genbank sequences with the cited accession numbers.
(PDF)
