# Supplementary figures and images for: Polyethylene glycol-functionalized poly (Lactic Acid-co-Glycolic Acid) and graphene oxide nanoparticles induce pro-inflammatory and apoptotic responses in Candida albicans-infected vaginal epithelial cells
Source: PLoS One. 2017 Apr 3;12(4):e0175250. doi: 10.1371/journal.pone.0175250 (PMC5378405; doi:10.1371/journal.pone.0175250)

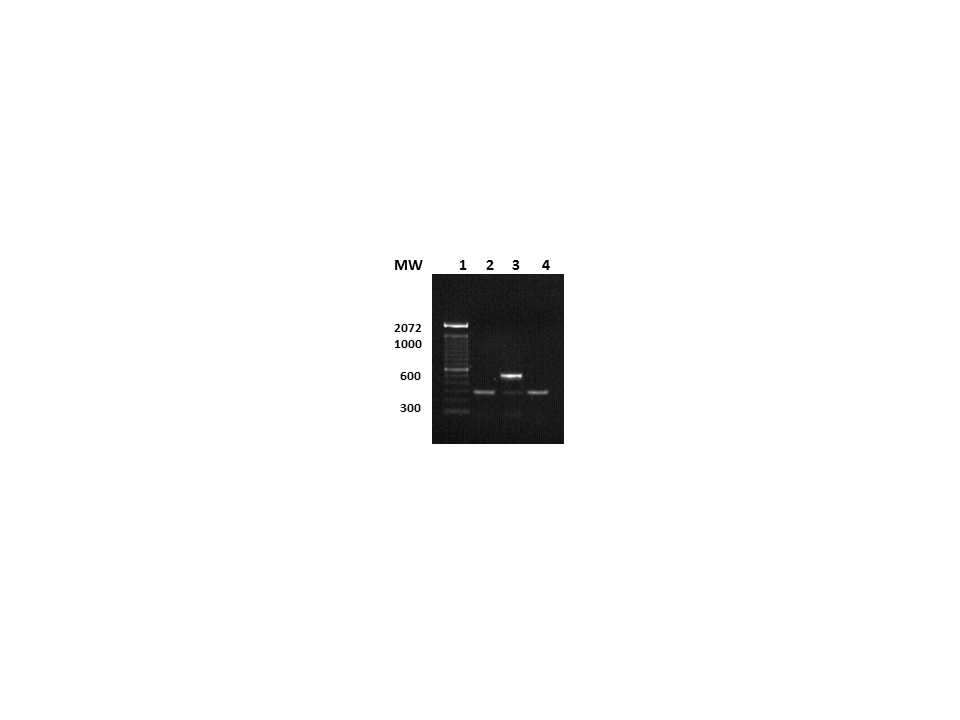

Supplement: S1 Fig — Evaluation of cell-line Mycoplasma spp. contamination status was measured by PCR. Lane 1 = Molecular Weight Marker, Lane 2 = negative control, Lane 3 = Positive control, Lane 4 = Sample of cells in culture media. (TIF) [file pone.0175250.s001.tif]
