# Supplementary material for: Polyethylene glycol-functionalized poly (Lactic Acid-co-Glycolic Acid) and graphene oxide nanoparticles induce pro-inflammatory and apoptotic responses in Candida albicans-infected vaginal epithelial cells
Source: PLoS One. 2017 Apr 3;12(4):e0175250. doi: 10.1371/journal.pone.0175250 (PMC5378405; doi:10.1371/journal.pone.0175250)
Supplement: S1 Table — The National Center for Biotechnology Information (www.ncbi.nlm.nih.gov) gene symbols and reference sequence accession numbers are listed for the mRNA molecules that were quantified by RT-qPCR using DNA primers synthesized by Qiagen, Inc., based on the Genbank sequences with the cited accession numbers. (PDF) [file pone.0175250.s002.pdf]

Table S1 List of genes analyzed by RT-qPCR

Gene Symbol	Refseq #	Gene Symbol	Refseq #	Gene Symbol	Refseq #	Gene Symbol	Refseq #
ACTB	NM_001101	DEFB4A	NM_004942	MAP1LC3A	NM_181509	RIPK1	NM_003804
AKT1	NM_005163	DHCR24	NM_014762	MAP1LC3B	NM_022818	RIPK2	NM_003821
ANXA2	NM_004039	DNAJA3	NM_005147	MAPK1	NM_002745	RNF7	NM_014245
APAF1	NM_001160	DNAJC3	NM_006260	MAPK3	NM_002746	RPL13A	NM_012423
AKT1S1	NM_032375	DRAM2	NM_178454	MAPK8	NM_002750	RPS6KA1	NM_002953
ATG3	NM_022488	DUOX2	NM_014080	MAPKAP1	NM_024117	RPS6KA5	NM_004755
ATG7	NM_006395	DUSP1	NM_004417	MAP3K1	NM_005921	S100A7A	NM_176823
B2M	NM_004048	EGR1	NM_001964	MAP3K7	NM_003188	S100A8	NM_002964
BCL2	NM_000633	ELK1	NM_005229	MBL2	NM_000242	S100A9	NM_002965
BECN1	NM_003766	ERO1LB	NM_019891	MCL1	NM_021960	S100B	NM_006272
BID	NM_001196	ESR1	NM_000125	MLST8	NM_022372	SELS	NM_203472
BMF	NM_033503	FADD	NM_003824	MMP10	NM_002425	SERP1	NM_014445
BNIP3	NM_004052	FKBP1A	NM_000801	MSRA	NM_012331	SLPI	NM_003064
CAB39	NM_016289	FOS	NM_005252	MTOR	NM_004958	SLC11A1	NM_000578
CAB39L	NM_030925	FOXI1	NM_012188	NAIP	NM_004536	SOD3	NM_003102

CALR	NM_004343	GABARAP	NM_007278	NFKB1	NM_003998	SQSTM1	NM_003900
CANX	NM_001746	GABARAPL1	NM_031412	NFKBIA	NM_020529	STAT3	NM_003150
CARD6	NM_032587	GABARAPL2	NM_007285	NLRC4	NM_021209	STK11	NM_000455
CARD16	NM_052889	GADD45A	NM_001924	NLRP1	NM_033004	SYCP2	NM_014258
CASP1	NM_033292	GAPDH	NM_002046	NLRP2	NM_017852	TCP1	NM_030752
CASP3	NM_004346	HERPUD1	NM_014685	NLRP3	NM_183395	TGM2	NM_004613
CASP7	NM_001227	HPRT1	NM_000194	NLRP4	NM_134444	TICAM1	NM_182919
CASP8	NM_001228	HSP90AA1	NM_001017963	NLRP6	NM_138329	TIRAP	NM_001039661
CCL2	NM_002982	HSPA1A	NM_005345	NLRP12	NM_033297	TLR1	NM_003263
CCL3	NM_002983	HSPA1B	NM_005346	NLRX1	NM_024618	TLR2	NM_003264
CCL5	NM_002985	HSPA8	NM_006597	NOD1	NM_006092	TLR3	NM_003265
CCL11	NM_002986	HSPD1	NM_002156	NOD2	NM_022162	TLR4	NM_138554
CCL19	NM_006274	IFNA1	NM_024013	NOS2	NM_000625	TLR5	NM_003268
CCL20	NM_004591	IKBKB	NM_001556	NUCB1	NM_006184	TLR6	NM_006068
CCR3	NM_001837	IKBKG	NM_003639	PARP1	NM_001618	TLR7	NM_016562
CCR7	NM_001838	IL37	NM_173205	PARP2	NM_005484	TLR9	NM_017442
CCT3	NM_005998	IL1R1	NM_000877	PLD1	NM_002662	TMEM57	NM_018202
CCT4	NM_006430	IL1A	NM_000575	PPARA	NM_005036	TNF	NM_000594

CD14	NM_000591	IL1B	NM_000576	PRKAA1	NM_006251	TNFRSF1A	NM_001065
CD27	NM_001242	IL3	NM_000588	PRKCH	NM_006255	TNFRSF10A	NM_003844
CD36	NM_000072	IL6	NM_000600	PTEN	NM_000314	TNFSF14	NM_003807
CEBPB	NM_005194	IL8	NM_000584	PTGES	NM_004878	TP53	NM_000546
CLEC7A	NM_022570	IL10RB	NM_000628	PTGS2	NM_000963	TRAF6	NM_004620
COLEC12	NM_130386	IL23A	NM_016584	PTX3	NM_002852	XIAP	NM_001167
CREB1	NM_004379	IL33	NM_033439	PYCARD	NM_013258	MAP3K5	NM_005923
CSF3	NM_000759	IRAK1	NM_001569	RAB24	NM_130781	SLC44A2	NM_020428
CTSB	NM_001908	JUN	NM_002228	RAGE	NM_014226	CD40	NM_001250
CXCL1	NM_001511	LAMP1	NM_005561	RPTOR	NM_020761	CD40LG	NM_000074
CXCL6	NM_002993	LGALS1	NM_002305	RELA	NM_021975		
CXCL9	NM_002416	LGALS3	NM_002306	RELB	NM_006509		
DEFB1	NM_005218	LY96	NM_015364	RICTOR	NM_152756		

The National Center for Biotechnology Information (www.ncbi.nlm.nih.gov) gene symbols and reference sequence accession numbers are listed for the mRNA molecules that were quantified by RT-qPCR using DNA primers synthesized by Qiagen, Inc., based on the Genbank sequences with the cited accession numbers.
